# The Future on Love and Business Organizing. An Agenda for Growth and Affirmation of People and the Environment (AGAPE)

**DOI:** 10.1007/s41463-021-00117-x

**Published:** 2021-12-02

**Authors:** Harry Hummels, Matthew T. Lee, Patrick Nullens, Renato Ruffini, Jennifer Hancock

**Affiliations:** 1 School of Business and Economics, Maastricht University, P.O. Box 616, 6200 MD Maastricht, The Netherlands; 2 Utrecht School of Economics at Utrecht University, Utrecht, The Netherlands; 3 Empirical Research, Human Flourishing Program, Institute for Quantitative Social Science, Harvard University, 1737 Cambridge St, Room 350, Cambridge, MA 02138 USA; 4 Leadership Ethics and Humanization of Society, University of Humanistic Studies, Utrecht, the Netherlands; 5 Evangelische Theologische Faculteit, Louvain, Belgium; 6 Departement of Law, Università Degli Studi Di Milano, C. Beccaria. Via Festa del Perdono, 20122 Milano, Italy; 7 Humanist Learning Systems, PO Box 256, Ellenton, FL 34222 USA

**Keywords:** Love, Agape, Business, Responsibility, Grammar

## Abstract

Business and love appear to have little to do with each other. We hold the opposite to be true if the concept of love in business draws from two corresponding grammars. This paper contributes to the ‘agenda for growth and affirmation of people and the environment’ (agape) in business. By focusing on the grammars of love and business we operationalize the concept of love in ways that business executives, managers and employees can understand, adopt, and implement. With references to the theory and practice of management and organizations, we aim to contribute to expanding the theory and practice of responsible organizations and their leaders caring for others.

## Introduction

The expansion and contraction of love is an ever-present dialectic in all organizations, including businesses. This involves the recognition that all human activity expresses some measure of love—or alternatively hate, or indifference. More importantly, organizations can more intentionally and systematically create an environment that promotes love. In this article, we invite business leaders to become aware of the dialectic and skillful in engagement with agape to foster the growth and affirmation of people and the environment. We start with an example from the construction industry and frame this engagement as a conversation involving the integration of the grammar of love with the grammar of business. In Sect. 2 of this contribution, we focus on the importance of love for human flourishing, followed by a philosophical foundation of love. Referencing the works of Fromm, Buber, Scheler, and Levinas we demonstrate that love is at the core of the logic of being and the recognition of the other as a person. In the words of Buber, the other is a ‘Thou’ and not an ‘it’ or an object in the hands of someone else. This foundation brings us to an exploration of ‘beneficial love’ in which we elaborate to ‘grammar of love’. Section 3 focuses on the grammar of business. Based on the exploration of the grammar of love, the question emerges how to best engage in a discussion on love in a business environment that is unfamiliar with this specific grammar? Moreover, businesses have their own grammar, aimed at achieving their mission in a constantly changing and challenging environment. A business grammar includes, among others, an understanding of the context in which a company operates, a focus on the company objectives, the organizational culture and its core values, the continuous interactions between a company’s leadership and its daily operations, participative decision-making, and the company’s economic, social, and environmental value creation. It also consists of inspiring stories that members of a business (community) find mutually meaningful and valuable to establish real communication. Drawing on lessons from the business and management literature we focus on the composition of the organization, its governance and decision-making processes, internal collaboration, and organizational performance. In Sect. 4 we draw some conclusions, after which we provide a tentative agenda for agape in business.

With this paper we aim to contribute to building an ‘agenda for growth and affirmation of people and the environment’ (agape). By focusing on the grammars of love and business we attempt to operationalize the concept of love in ways that business executives, managers and employees can understand, adopt, and implement in their daily business operations. The agenda is more comprehensive, however, than we can elaborate on in this initial paper. Based on both grammars we intend to pursue our path towards promoting beneficial love in business organizations through further explorative research into a more elaborated theoretical framework, tools and instruments and an overview of best practices – and we invite others to do so as well. We start this paper with an introduction to the challenging issue of love in business.

## Attunement: Love as a Business Practice and an Agenda for Future Business Organizing

In the late 1920s, in the years leading up to the Wall Street Crash, New York faced an enormous construction boom. In those days the building industry was known for bullying employees, paying extremely poor wages, and foregoing on safety precautions. These practices resulted in minimal productivity, resentment, and pilfering. How different was the situation of Empire State Inc., the consortium of investors responsible for the construction of the Empire State Building? It obtained the land just months before the collapse of the stock exchange and, despite the poor economic foresight of a bear market for office space, the owners decided to contract a building company that invests in the well-being of the construction workers. The company doubled salaries, covered lost hours when weather conditions prevented workers from climbing the building, and took care of subsidized meals. The result: from start to finish it took less than thirteen months to construct what at that time was the highest building in the world. An impressive achievement, even by today’s standards (Bodanis, 2020).

It may sound odd to think of the conduct of the company that constructed the Empire State Building in terms of beneficial love for their workers, but that is precisely the statement we want to make.^1^ The mission of a business organization can be achieved in various ways, as the example of the construction boom in New York suggests. One can treat employees as means in the hands of management to do a needlessly risky job for which they receive scant payment. One can also treat employees as ends in themselves, as humans who are paid due respect and whose dignity (and physical safety) is acknowledged by their employers. The construction of the Empire State Building suggests that organizing, as the practice of bringing together humans and non-human resources for a common objective and facilitating their interaction, may be more efficient, effective, and humane if employees are treated with love. That does not mean that business leaders who treat others with respect and recognize their dignity are necessarily naive and doomed to fail in achieving their business objectives. To the contrary, we argue that beneficial love offers advantages for employees, for the business, for the broader society, and for the natural environment.

This paper contains an agenda for the promotion, adoption, and implementation of love as a practice of organizing for human flourishing and for the protection and thriving of our natural environment. This *Agenda for Growth and Affirmation of People and the Environment* (AGAPE^2^) starts with a reflection on *the meaning and grammar of love in processes of organizing*. What does love mean and how can it support company leaders aiming to conduct their business with respect for employees, clients, suppliers, business partners, owners, nature, and the communities they affect – while at the same time achieving their mission and objectives? A recognition of the meaning of love can even lead to broadening the organization’s mission or of the ways in which the mission is achieved.

At the same time, an effective *conversation* with business executives and managers on the meaning and value of love in organizing requires a deep understanding of business practices. Building an agenda of love and organizing therefore requires understanding the grammar of business. This second grammar will allow company leaders – and by this we mean all people within an organization who are influential, regardless of their position in the formal hierarchy – to reflect on creating and maintaining a business practice that supports and enhances collaboratively *living love* in daily business life. It deals with the structures, procedures, and the culture of decision-making. What does love require in terms of, for instance, participatory decision-making to establish and maintain a love enhancing business climate?

Why did Empire Building Inc. deviate from the commonly accepted practice of poorly treating workers in the construction sector? Why did it, among other things, pay more, compensate workers for lost hours, and provide decent meal services? The business sense of treating workers with dignity and respect is demonstrated in the pace with which the building was constructed. This raises the question of why so many business leaders fail to see past the limited set of ‘hard’ financial and economic incentives and promote human flourishing, dignity, and respect for others in achieving the business objectives. As a consequence, this paper reflects on motives and drivers associated with love as a business practice. The example of Empire State Inc., and other companies in a variety of sectors, shows that exerting love in business is possible across the board for all companies. Inspiring examples of companies that lead the way often provoke more positive responses than just telling corporate executives what to do. Therefore, and to support executives and managers in building their own agenda for love and organizing, our next step will be to focus on best practices that master the grammar of love and succeed in integrating it in the grammar of business. Finally, an agenda for the future of love and organizing should focus on the dissemination of the interacting grammars to enhance its adoption and promote the emergence of a community of business organizations living love in their daily operations. The *conversation* this requires between different stakeholders will often not be easy. They may speak a different language, use different frames of reference, and operate in different environments that sometimes limit the room for maneuvering and experimentation with new concepts and ideas.

Conversations on love and organizing often have traits of an Argentinian tango. When in harmony, it excites both dancers and spectators. It is passionate, skillful, and beautiful to observe. But not all dancers are professionals. Some are just starting to master the basic steps and have difficulty in responding to one’s partner. It leads dancers to be out of tune and to step on one another’s toes, resulting in the interruption of the tango. Engaging in conversations on love as a practice of organizing faces more or less the same difficulties. Most business executives and managers have not yet mastered the tango of love in business. The first move is then to engage with them and try to excite them about the beauty of the dance and maybe show them some examples of other businesses. What environment have they created for their internal and external stakeholders to make their first moves and to demonstrate love in organizing as a business practice. The challenge lies in *attuning different ideas* about what constitutes a good life, overcoming different knowledge-bases and skill sets, acknowledging different paces of life, and appreciating different conceptions of success. Our challenge in this paper on the agenda for growth and affirmation of people and the environment is that we won’t be giving executives and managers the answers, we are inviting them to engage themselves in the discussion and define the answers for themselves.

An agenda for growth and affirmation of people and the environment consisting of these elements (grammar of love, grammar of business, why love, best practices, and dissemination), provides useful guidance to start a discourse on the role of love in business. Its ultimate objective is not just to engage in a conversation on love, but to raise awareness and promote designing, developing, and implementing an environment in which love is practiced for the good of the business, of humans involved in the business and of humanity and the natural environment in which we live and work. Love is not just a word or a concept, but also a way of understanding reality and a practice.

## The Grammar of Love

“Get real”. It is a phrase commonly heard once you start to talk about love in a business environment (Sisodia et al. 2014). Love is not a word that one often encounters in business conversations as it fails to clarify its immediate relationship with the mission of a business and the executives, managers and employees working in that business. The discomfort that many businesspeople experience when the conversation shifts to love, often comes from a different understanding of what the concept could truly mean within the context of leadership and organizing in business. In this section we will elaborate beneficial love as a productive concept that enhances human flourishing while at the same time contributing to the enactment of the mission as defined by the organization. First, we provide four arguments why the grammar of beneficial love requires our attention for understanding the practice of leadership and organizing in a business context. Then we give a brief description of the concept of beneficial love.

But first it is necessary to note that various types of love are always evident whenever human beings interact with each other. Forms such as affection (storge), friendship (philia), and passion (eros) are considered good in the right situation, at the right dose, and for the right purpose, but without a more extensive expression aimed at human flourishing or well-being (agape), such love tends to produce problems (Lewis 2012). One serious limitation of non-agapeic forms of love is their tendency towards selfishness, insularity, and the requirement of reciprocity. The “tragedy of tribal altruism” is one especially pernicious manifestation that occurs when a group loves its own members so intensely that ill-treatment of an out-group is justified and encouraged (Sorokin 2002:459). Contrary to common usage, a healthy understanding of agapeic “love” is inconsistent with harmful passion, permissive sentimentality, and hatred towards an out-group; it instead requires a strong sense of the infinite value of all people and the natural environment, a “sense of a common bond with humanity” that is the “heart of altruism.” (Xi et al. 2017:67) After all, unrequited passionate love can quickly turn into love’s opposite: hatred, indifference, and resentment. Human behavior is often motivated by one or more of the types of love. Whether or not the label of “love” is expressly used in business contexts, few business leaders would want to be labeled as unloving or uncaring towards the well-being and flourishing of employees, customers, society, or the environment. The expansion and contraction of agapeic love is an ever-present dialectic in all organizations, including businesses. Organizational actions can reflect a healthy form of agape that is (re)generative for people and the environment, or these actions can foster diminishment in ways that are best described as indifference or perhaps even hatred. Often, the same collective action can express both the promotion and the reduction of human and non-human well-being and flourishing. One of our goals is to make this fact more apparent so that leaders might create a context for agapeic love and encourage actions that more intentionally and skillfully express this love for individuals, groups, and non-human life.

### Four Reasons for the Grammar of love as the Foundation of Humanistic Management

The first reason for the grammar of beneficial love is the current paradigm shift within economics, especially concerning the understanding of the human person. *Homo economicus*, the calculating person merely driven by prudential self-interest, is an outdated architype that does not reflect the full range of human motivations or the full experience of the flourishing life (Guillen et al. 2015; Van der Weelen, 2017; 2019; Guillen, 2021). Nor does it reflect the inherent interconnections between human flourishing and broader environmental health (Raworth 2017). Failure to fully connect with these motivations and aims may partly explain why surveys reveal that only a minority of workers are “engaged,” in the sense of being “highly involved in, enthusiastic about, and committed to their work and workplace.” (Harter 2020) Worldwide, the percentage of engaged workers over the past decade has ranged from 12 to 22%. In the U.S. the range goes from 28 to 38% (Wigert et al. 2021). Clearly, there is room for improvement. Neo-liberal myths notwithstanding, the human person is deeply relational being (Van Nes et al. 2021). What we call ‘humanistic management’ refers to a broader anthropology based partly on evolutionary biology embracing all fundamental drives of people. We have a drive to acquire, to defend, to comprehend and to bond. These four drives need to be balanced to create more human dignity in business (Pirson 2017). It is the drive to bond, the fact that we are a *zoon politicon*, as Aristotle argued, which makes the grammar of love essential for the promotion of human flourishing.

The purposes of business, to the extent that they engage with deeper human motivations, have evolved to include the creation of multiple values. Beyond generating profit, these motivations encompass addressing social, societal, and environmental challenges. Businesses are an important platform for addressing society-wide sustainability and improving wellbeing. There is broad awareness about the role of business for the common good. Business strategies such as Corporate Social Responsibility and Triple Bottom Line are now widely accepted and reflect a somewhat enhanced moral awareness, although just how much is quite contested. A good example is provided by B-Lab, a network of certified B-Corps that sets a high standard. This multiple value creation requires a change in business modelling. What is often overlooked in this paradigm shift toward a more sustainable and inclusive economy is a sense of responsibility. An experience of connectedness is the essential condition to make multiple value creation happen. Such connectedness helps to create (re)generative, rather than merely efficient or sustainable, processes (Reed 2007). The (re)generative processes are vital in our era of extraordinary environmental degradation, epidemic human disengagement, burnout, and multiple systemic disconnects that reflect a state of “organized irresponsibility, collectively creating results that nobody wants.” (Scharmer and Kaufer 2013:1) They are also essential in generating a new way of doing business that respects and nurtures humans and the natural environment as valuable in themselves.

The second reason is that the grammar of beneficial love deserves a prominent place in the values, the ethics, and the behavior in business. Beginning in the 1960s, in response to many disastrous scandals in finance, industry, and business, and to reflect on the role of business in society (Bowen 1953; Carroll 2008), courses on ethics became a required part of the curriculum in most business schools. Indeed, it is difficult to identify a business activity that does not have an ethical dimension, regardless of whether the issues are related to human resources, intellectual property, consumer protection, marketing, accounting, worker safety, or environmental impact. Ethical leadership is an especially prominent topic, with recent models of leadership such as transformational leadership, servant leadership, and authentic leadership constructed as the very heart of leadership. To give one prominent example, based on Aristotelian ethics, a true leader is understood as respecting others, serving others, demonstrating justice, manifesting honesty, and building a healthy community (Northouse 2016). On this view, to build a community of belonging and collaboration is a business responsibility and does not involve using people as means for an end. Leaders generate positive psychological capital in the workplace increasing efficacy, optimism and resiliency (Luthans et al., 2015). Authentic leadership embraces the importance of relationships and connectedness (Gardner et al. 2011). A grammar of love contributes to this emergent understanding of leadership, and it would be helpful for leaders and all stakeholders if we collectively became more intentional and skillful in our expression of love.

The third argument for the importance of the grammar of love is somewhat more abstract, yet not less significant. What is ‘good business’ or ‘good leadership’? Often morality is merely understood in terms of constraints: what we should and should not do. A focus on the constraints of law, ostensibly creating a level playing field of free trade, reflects a reductionistic, fragmented, and dehumanizing view of ‘ethical business.’ In line with the philosopher Paul Ricoeur (1992:169), we make a distinction between ethics and morals. Ethics is about (the reflection on) the aim of our praxis and morals about the obligations of our praxis. Ethics in business is based on the aim of an accomplished life and morals about the norms, obligations, and constraints. The one is teleological and the other is deontological. According to Ricoeur, ethics has primacy over morality, what is the aim over the norm. The grammar of love embraces both. It gives a higher purpose and generates moral principle. The primal focus on aims and human flourishing and secondly a deontology of love is provided. This requires, according to Ricoeur, a personal as well as an institutional challenge. Therefore, Ricoeur defines ethics as “aiming at a good life lived with and for others in just institutions” (Ricoeur 1992:172). The grammar of love is not only a private issue, it is crucial for just institutions (Ricoeur 1995.

The fourth reason for the grammar of love has to do with a new type of logic and language that needs to be developed. Ethics is embedded in identity, narrative and language. To give an example from the world of investment baking and the financial crash of 2008, Joris Luyendijk, a journalist from *The Guardian* and cultural anthropologist, interviewed over a hundred investment bankers in the London financial district known as the ‘City” (Luyendijk 2015). These high-risk and highly-paid individuals exist within a culture in which the institutional logic encourages others to be treated merely and sometimes only as means to an end and a code of silence about ethical issues prevails (cf. Jackall 1988). The global financial crisis of 2008 was a predictable outcome of such institutional arrangements and future scandals are virtually certain to occur. But according to Luyendijk, the cause of this crisis, and others before it, is not only traceable to ‘greed’. Financial incentives do play a role, but in addition, the industry had come to rely on sophisticated algorithmic models to the point that investment products are not only incomprehensible to outsiders but to many bank managers and traders as well. It resulted in short-term thinking focused on shareholder value and personal bonuses combined with a “cloud of self-delusion” (Luyendijk 2015:225). Under these social conditions, it is rare to talk in terms of purpose or meaning. It was not a part of the predominant grammar. One insider remarked that, “there’s a particular kind of insecurity to many bankers, a form of neediness and a deep desire to compensate for something. The absence of love, perhaps.” (Luyendijk 2015:223).

### Moving from the Logic of Having to the Logic of Being

The deeper issue represented by these bankers is what the psychoanalyst Erich Fromm described as a world that is built mainly on the logic of having and not – or significantly less – on the logic of being (Fromm, 1976). These are two fundamental forms of experience, and both are necessary for human flourishing. The logic of having refers to instrumental reasoning and is oriented on progress and prosperity. The main tenet of Fromm’s theory is that we are increasingly driven by our having mode of existence at the expense of our being mode of existence. The dominant having-oriented logics of being creates a pathological society, addicted to pleasure-seeking. Consumerism reduces the world to objects that match with desires, in order to use them without being deeply interested in them. Happiness becomes an object to be achieved: I am = what I have and consume. The idiomatic framing of having looks at people as consumers or producers (human resources). This type of logic creates toxic forms of self-identity and language. Excessive consumerism and selfish hedonism leads to a pathogenic syndrome of boredom, chronic depression, fear and loss of consciousness, associated with the desire for belonging through demonstrative consumption, self-improvement, and image cultivation (Fromm 1959). This contributes to a “mindset of maximum ‘me,’” and an “ego-system” that is unfavorable to inner peace, harmonious social relations, and a healthy natural environment (Scharmer and Kaufer 2013; Xi and Lee 2021). We do not propose the complete abandonment of the logic of having, certainly not in the context of business. The business sector makes significant contributions to the quality of life and the protection of humans against the forces of nature. But a greater emphasis on the logic of being, in the context of a grammar of love, has the potential to address the root causes of the habits of extraction and disconnection that are unsustainable and unjust for society, the economy and the planet to thrive. In describing the forms in a business context, we recognize that concepts such as the good life, flourishing, and love itself are all contested.

More fundamentally, love as a logic of being and not only as having is a particular way of relating. We can start our understanding of beneficial love with the analysis by the Jewish philosopher Martin Buber (2010). In a business context, many relationships are impersonal and transactional. These I-it relationships are typical for a focus on having, which Buber acknowledges are unavoidable in large-scale societies. But such relationships, according to Buber, can be dehumanizing when overused and when they foster violent ways of relating. In contrast, I-Thou relationships reflect the logic of being. The Thou is not an object that we can have. Instead, we value the Thou as a person – as a sacred subject rather than profane object – and we connect with or are attached to that person. It is the melting of the between, the immediacy of meeting. According to Buber human life approaches its fulfilment, its redemption, to the extent that the I-Thou relation becomes strong. Much business (and non-business organizing) displays an I-It relationship. The grammar of love seeks to infuse the I-Thou dimension into business relations, whenever possible (Ossewaarde-Lowtoo 2018). Again, the issue of balance is an essential consideration. As Buber recognized, it would not be possible, or even desirable, to eliminate all I-It relations.

As an enhancement to the frameworks developed by Buber and Fromm, we add Scheler’s (2007) understanding that love is not just a moral commandment. It is foremost an epistemological category, a way of seeing, understanding, and discovering true values (Scheler 1973; Nullens 2018). This relational viewpoint is only recently beginning to take root in cognitive science, which like business, has reinforced the cultural project of “dividing and conquering.” (De Jaegher 2019) In contrast to the analytical way of knowing the world by carving it into discrete entities in ways that deemphasize the importance of interconnections and love, an epistemology grounded in love tends to promote symbiosis and mutual benefit, rather than separation. The relevance of our way of seeing and understanding the world outside us, and in particular other human beings, is also addressed by Emmanuel Levinas. According to Levinas there is no ‘universal other’; there is only the particular other. The other appeals to me through our face-to-face encounter (Levinas 1969). Yet business systems—like other large-scale, bureaucratically organized processes—tend to reduce the other to sameness. Under such conditions, executives and managers may not see employees, customers, or stakeholders as individual persons with dignity and infinite worth, but instead see employees as wage laborers selling themselves as mere commodities in return for a financial compensation, customers as sources of revenue to be exploited by manipulative advertising, or in the case of stakeholders, as sources of potential friction to be neutralized. In other words, the executive sees the other through his or her own eyes—resulting in totalization.^3^ Love, on the contrary, is open to the uniqueness of the other, it is a way of de-totalization (De Jaegher 2019). This is not the case only for individuals, but also concerns loving ways of knowing and relating to the life-sustaining ecosystems of the world as well (Reed 2007).

### Beneficial Love: a Love that Works in a Business Setting

The above-described relevance and philosophical underpinning of love suggests that it is important to inquire into how business executives and managers perceive themselves and others, and how they perceive the meaning and value of love. We argue that a productive way to have a conversation about love with executives and managers is to use the concept of beneficial love (Hummels 2022). This builds on previous applications of both agape love and healthy, mature love to organizations. The concept of beneficial love contains three elements:

- 1) it is comprised of constituents that promote flourishing for others and self,
- 2) these elements are arranged with practical wisdom according to a unifying grammar, and.
- 3) the resulting arrangement signifies a life-affirming story.

The first element, while recognizing that some popular understandings of love include constituents such as jealousy, possessiveness, harm, and the like, focuses on the components that promote flourishing (VanderWeele 2017; 2019). At the individual level, these include: patience, truthfulness, kindness, respect, trust, generosity, empowerment, warmth, understanding, and many others (Lee 2022). Such love is fundamentally relational, as it is necessary to behold the other as a Thou rather than an It in order to know what might promote their complete well-being. In a business environment, for example, the relationship between a leader and her or his team/unit/organization can fall along a continuum between It and Thou, involving some combination of the logics of having and being.

How the constituents of love are arranged is shaped by the second element: a living grammar that organizes words so that we can construct proper sentences, thereby enabling effective communication. Love, in this respect, is a unifying grammar that, in a healthy and productive manner, contributes to the good of the self and of the other and unites them. An unhealthy grammar combines the constituents of warmth and violence, for example, in a romantic relationship that is both controlling and passionate and ultimately not loving in the benevolent sense we have in mind. The mere presence of constituents of love is therefore not sufficient to qualify as beneficial love.

Therefore, the third element frames beneficial love as a *latent construct* that emerges when the grammar combines constituents in a life-affirming way in specific relationships that unfold over the life course in developmentally appropriate ways. The construction of constituents, the rules of the grammar, and the meaning of the story are always structured by social institutions and culture, but common elements have been empirically identified as important across social groups. The story of love is most life-affirming when it promotes the deepest flourishing of all people, living things, and life-sustaining systems. By connecting love to flourishing at multiple levels of analysis, we will necessarily engage with justice, fundamental dignity, and above all else, (re)generation of the worth of the other as a person and the recognition of the intrinsic value of nature. The latter is required because the baseline has been a pattern of harmful, extractive, disconnected, and degenerative ways of knowing and relating to self, others, and ecosystems that require healing and reconnection (Reed 2007; Scharmer and Kaufer 2013; Laloux 2014; Sisodia and Gelb 2019). In other words, love is much more than sentimentality, although open-hearted warmth in relationships is both desirable and beneficial. (Kongtrul 2018) Love, as we conceive of it, refashions flourishing in ways that transcend the logic of having and I-It relations, the exploitative “unending pursuit of economic growth and… the seemingly endless quest for consumer goods” (Carlisle et al. 2009: 1556) that characterize much contemporary business activity. Explicit attention to power is necessary. As Martin Luther King, Jr. (1967) famously put it:

“What is needed is a realization that power without love is reckless and abusive, and that love without power is sentimental and anemic. Power at its best is love implementing the demands of justice, and justice at its best is love correcting everything that stands against love.”

It may be true that our concept of love does not align with institutional logics such as the one found in London’s financial district that we described above. But the narrative of love we have in mind is not designed to nudge the current system to engage in current activities in an improved way, but rather to encourage a shift towards more (re)generative awareness and activities. In other words, away from the perspective of “effectiveness and efficiency—‘doing things better’” and towards the perspective of “doing better things” and “seeing things differently.” (Reed 2007: 675–676) Early adopters in business have made important strides in this direction (Hummels 2022). By integrating the grammar of love and the grammar of business, we aim to provide realistic guidance to business leaders, with the full recognition that many contemporary business practices are inherently degenerative and will need to be wholly abandoned. Business itself can thrive by connecting more intentionally to a life affirming narrative of flourishing for all. For existing businesses to turn the tide, the grammar of love needs to be explained in terms that executives, managers, and employees understand and are able to apply in the context of their daily business. It requires academics and practitioners, arguing for an agapeic turn, to develop their grammar in a way that they can “swim with the sharks without being eaten alive” (Sisodia and Gelb 2019:27).

## The Grammar of Business

If businesses are sharks, proponents of an agapeic turn not only need to develop their own grammar in ways that prevent them from becoming an easy quarry, but they also must understand the grammar of business. They should familiarize themselves with the mission and objectives of the business, its competitive environment, its tradition, and the ‘ways in which business is done around here’. We start this section with the story of Starrett Brothers Inc. and the construction of the Empire State Building, followed by a brief outline of the grammar of business and lessons that we can draw from the management literature.

### A Loving Construction Company

Paul Starrett was born in 1866 in Lawrence, Kansas, as the son of Presbyterian minister, farmer and lawyer William Starrett and his wife Helen Ekin, a Quaker teacher, journalist, and editor. When, in June 1928, he met with Empire State Inc. investor John Jakob Raskob as head of Starrett Brothers Inc., he already had a reputation in New York and other major cities.^4^ Starrett had been responsible for the construction of the Flatiron Building, Penn Station and H.R. Macy’s Department Store in New York, The Lincoln Memorial in D.C., among other buildings, as well as the Ford River Rouge plant – in 1930 the largest industrial complex in the world. Despite his experience, Starrett knew that the Empire State Building was something else. From the start – tearing down the old Waldorf Astoria – to finish, the construction of what was going to be the world’s largest building at the time had to be completed in thirteen months. What made it challenging is that neither the Starrett Brothers, nor anyone else, had the equipment to construct a one hundred-and-two-story high building, nor sufficient manpower to do the job. It was his honesty that got him and his brothers the assignment. It was his ‘fairness’ towards his workers that resulted in the building being completed on time, as David Bodanis argues. To provide some inside in the New York building industry, Bodanis (2020:61) sketches the following picture:

“Workers in the years leading up to the Empire State’s construction had generally been treated awfully, with wages kept low, at best $7 a day for labourers. If they wanted a hot meal during the day, they lost pay for the time it took to get down to the ground, find a good food wagon or diner, and then climb back up. Safety laws scarcely existed, and there were numerous deaths, and the perilous cranes went up.”

Starrett Brothers worked with a different philosophy. The company made sure that dedicated safety squads took all necessary precautions to minimize injuries or fatalities. It also paid its workers their full salaries on days strong winds would prevent the men from going up:

“Oh, and Starrett was going to more than double basis wages for all workers on the site, making the rate $15 a day – and there would be good-quality, subsidized restaurants on floor after floor of the building as it rose up.”(ibid., 2020:61)

These measures did not harm Starrett’s bottom line, nor did it increase Raskob’s investment in money or time. On the contrary, the construction company was known for its swift and economic execution – including money-saving efficiencies.^5^ His policies resulted in less accidents, idleness, employee turnover, and other labor related costs. On 11 April 1931 the building officially opened its doors.

It is very unlikely that Starrett used the word ‘love’ in the context of his business, even though he adopted the *concept* of beneficial love. Starrett was a grumpy man, he hardly ever smiled and wasn’t known for his friendliness or good humor. Bodanis, therefore, prefers to speak of Starrett’s fairness, generosity, and benevolence towards his workers and of the constructor’s sense of decency, rather than love or liking. The workers reciprocated what they received from the company by showing gratitude. By being treated fairly and feeling appreciated, they worked harder and were motivated to deliver a first-class job. Also, they used their creativity and came up with all kinds of ideas that would improve or speed up the construction process. Nevertheless, Starrett created a process that allowed him and his right hand, John Bowser, to keep oversight and assure that everyone knew that he was strictly aware of what was going on. The construction industry was known for fraud, theft of equipment, idling, and so forth. Starrett therefore ‘gave but audited’. Bowser commented on his hiring staff to physically visit each man on the construction site: “This method takes away from the foreman the temptation of favoring nonexistent accounts”(Bodanis 2020:67). Also, to assure that inventory would not walk away, he checked whether equipment remained where it was supposed to be. Given the context in which Starrett operated, his behavior was remarkable and displayed what we call ‘love for his workers’ – even though he did not use the term. Why then do we think the concept of love adds value to our daily conversations in business? In the end, we hope to demonstrate that introducing the concept helps to create a more mature, humane, and meaningful context in which people realize so much of their potential as human beings, while at the same time contributing to a successful business venture and a more sustainable, just and inclusive society and planetary ecosystem.

### Understanding the Grammar of Business

Paul Starrett was not an exceptional business person. He operated a construction company, albeit in a different, more humane way than the competition does. Nevertheless, Starrett Brothers Inc. also thinks in terms of strategy, product offering, cost accounting, operational planning, procurement, making profits, getting the right people for the job, and customer relationships. So how does this grammar of business and organizing relate to the grammar of love? Introducing the latter grammar means we must have a convincing answer to the question: ‘What should I do as (C-level) general manager, sales manager, procurement manager, HR manager, financial manager, et cetera, to introduce, implement and preserve agapeic, beneficial love?’ In other words, we have to clarify what it means to make an ‘agapeic turn’ in a business context from what we previously referred to as shift from I-it relations to I-Thou.

The reference to Paul Starrett in a *conversation* on the meaning and value of the concept of love in processes of organizing starts by acknowledging the success of his business venture and an understanding of what made Starrett Brothers Inc. stand out of the crowd. Just like any other organization the construction company can be seen as ‘a temporary sediment of ongoing processes of interaction in and between dynamic groups of people to achieve certain agreed upon objectives’ (Weick 1979; 1995). To construct the Empire State Building it brought together numerous people and business resources to erect the edifice. Understanding what love means in this business context requires understanding a company’s commitment to return profits that sustain the long-term viability and flourishing of the company. The conversation then continues when it allows company leaders to reflect on creating and maintaining a context that enhances the mission and the business objectives and to act on it, while at the same time collaboratively *living love* in daily business life. The essential point to make is that business leaders have a responsibility and an opportunity to create an environment – including a culture, decision-making processes, performance review processes, meaningful jobs, recognition of the individual’s contribution to collective results, etc., opportunities for personal development and growth, etc. – in which employees, clients and other stakeholders can flourish and thrive. Living love then refers to listening to and respecting others as Thou in the governance of the organization, its culture and procedures, and the decision-making in perpetual processes of organizing.

In the context of business, what is required is the alignment of the goals, the culture, the decision-making, the outputs and outcomes and the continuity of the business with the promotion of “the deepest flourishing of all people, living things, and life-sustaining systems”. As a consequence, love in business is not the same as promoting a culture of charity (Dees 2012), modern generosity (Bodanis 2020), or CSR phrased differently. It also is not the same as a call to interpersonal kindness, empathy, and compassion. Although these elements are relevant and may be beneficial to the business, they miss the point. Following the second element of beneficial love, we can understand the wisdom of and the interaction between executives, managers, and employees to achieve the business objectives within an environment that is fundamentally aligned with the promotion of human flourishing and respect for the societal system of which businesses are a part. At its core, beneficial love expresses a commitment to the well-being of the stakeholders as subject – instead of as objects – while trying to run a business and return a profit. Love in business balances both sides of the equation, instead of choosing for one side. “Choosing between caring for others and self-interest is like choosing between breathing in and breathing out”, as Sisodia and Gelb (2019:19) remark. What then is the lingua franca of the business community that allows for introducing a grammar of love and how can it help executives, managers, and employees to create an environment in which individuals, groups and the organization as a whole can thrive within the societal context in which it is operates? What does it take to create policies, practices and resources that support the introduction, implementation, and preservation of human flourishing? What do executives have to promote and what do they have to refrain from or change in the organization to live love? Perceiving companies as ‘processes of organizing dynamic interactions between individuals and groups aimed at a certain agreed upon objectives’ requires an analysis of what makes them successful, both operationally and financially, as well as in terms of (their commitment to) the flourishing of individuals and groups in their social and natural environment. In other words, we have to establish the connection between the existing literature and practice of successful businesses and the concept of love in business.

### Integrating the Grammars of Love and Business: Lessons from the Literature

Looking at the business literature over the last 25 years, we can distill several core elements that are relevant for businesses to become successful as excellent, lasting, living, individualized, great, sustainable, teal, healing, fair, or mutual companies (Peters and Waterman 1982; Collins & Porras 1996; De Geus 1997; Ghoshal and Bartlett 1997; Collins 2001; Laszlo 2003; Laloux 2014; Sisodia and Gelb 2019; O’Toole 2019; Bodanis 2020; Mayer and Roche 2021) – all having some potential connection with beneficial love. What are these core elements of success that can help executives if they set course for a company that lives love^6^ and academics to better understand business leaders? All currents in management literature – from Mary Parker Follett, Chester Barnard, Peter Drucker and more recently Jim Collins or Frederick Laloux – explain the principles of management and provide some conception of what turns a company into a successful company. Most management authors are in agreement on the importance of following elements:

1. The composition of the members of the organization
2. The organizational governance and decision-making
3. The collaboration between the organization’s members
4. The organizational performance in terms of outputs and outcomes

In the remainder of this section, we will elaborate each of the elements. In general, all theorists would subscribe to the conclusion of what O’Toole describes as ‘enlightened capitalists’:

“none was primarily a philanthropist; none started with the intent of using his or her business to do good work (…); all were committed to the development of employees; and all were dedicated to ethical dealings with their constituents (specifically, by treating them with respect).” (O’Toole 2019:428)

#### Getting The Right Organizational Members Onboard

Focusing on the business and management literature of the last two-and-a-half decade, there seems to be one thing that binds most scholars: the essential contribution of committed and motivated employees to the (potential) success of a business. It is about ‘putting people first’. “What the hell else is there?”, Tom Peters asks (quoted in Sisodia and Gelb 2019:xvi). Or in the words of former GE CEO Jack Welsh:

“The talents of our people are greatly underestimated, and their skills are underutilized. Our biggest task is to fundamentally redefine our relationship with our employees. The objective is to build a place where people have the freedom to be creative, where they feel a real sense of accomplishment – a place that brings out the best in everybody” (Quoted in Ghoshal and Bartlett 1997:8)

Having said that, not everyone is qualified or motivated to contribute to the success of the business. As Collins observes: “The executives who ignited the transformations from good to great (…) first got *the right people* on the bus (and the wrong people off the bus) and then figured out where to drive it.” (Collins 2001:43) The striking transformation of one of the most notorious prisons in the world underscores this point. When she assumed leadership of Tihar Central Jail in Delhi, India, Kiran Bedi (2006) found a violent, hopelessly overcrowded, and deeply corrupt institution. Many of the guards and supervisors were ingeniously unscrupulous. Worse, the legal system would not permit her to fire these individuals, despite their criminal activity. She eventually had to pay them to stay home in order to move forward with her compassionate reforms. As a result of her visionary leadership, Tihar Central Jail made remarkable progress in the direction of greater flourishing for prisoners and staff alike, with the result that Bedi received a medal of honor (the Ramon Magsaysay Award) and widespread international acclaim for her transformative work. A particularly astute observer described her “management style” that was “so wildly transformative” as being derived from “a single quality”: love. (Maparyan 2012:224) This case demonstrates that “love is a powerful social change technology…. Kiran Bedi entered Tihar Jail with this kind of love and, with this energy, made it possible for others to become bearers of this kind of love as well.” (Maparyan 2012:227) To reflect this orientation, she painted the word “Ashram” over the word “Jail.” Within this loving context, drug-addiction declined while “self-actualization” improved, as did “health and morale.” (Maparyan 2012:213, 221).

Figuring out who the right people are is done collectively. Being the right person, Collins continues, does not mean that you always have the best knowledge or skills. “Whether someone is the ‘right person’ has more to do with character traits and innate capabilities than with specific knowledge, background, or skills.” (Collins 2001:64) It often takes time to get the right people on the bus, but if you finally have them, they will be self-motivated (ibid:74). It is all about the ‘fit’ between the organization and the individual. If there is a fit the individual may “find it a great place to work”, if not “you will likely […] be expunged like a virus. It’s binary” (Collins 2001:9). Organizations can increase the chances of a fit between the current employees and a new hire by involving the existing colleagues in the selection process, starting with jointly developing the profile of their new colleague (Laloux 2014:154). Several organizations, ranging from large corporations like Semco to smaller firms like Viisi implemented this idea.

Viisi is a small but rapidly growing mortgage advisory agency in The Netherlands. The organization takes ample time for the selection of new hires and involves many ‘Viisionairs’, particularly those who will closely work with the newcomer. They have to consent to hiring their future colleague. Every new hire starts an extensive onboarding in the company’s happiness factory – to become acquainted with the core processes in the organization and its culture.

#### Organizational Governance and Decision-Making

Successful organizations that stand the test of time emphasize governance that is based on a sensitivity towards the external environment, the needs and interests of stakeholders, objectives that go “beyond just making money” (Collins and Porras 1996:8), transparency, and leadership as a “collective capacity in a system” (Scharmer and Kaufer 2013:112). Collins already emphasized the relevance of leadership that involves others by asking questions – not to illicit accountability within the organization, but to gain understanding. It is essential, the author continues, to revert to informal meetings to learn what is happing in the organization and the world outside. Great companies, according to Collins, engage in dialogue and create a climate of debate. To an important extent, they decentralize their decision-making power. De Geus observes in this context that the concept of decentralization is a twentieth century invention. In previous centuries business leaders would have used a different term: tolerance. Tolerant companies allow their members to develop initiatives, to go beyond existing boundaries, and to pursue new objectives – all within a certain bandwidth. It helped them to stretch “their understanding of possibilities” (De Geus 1997:7), but also to remain ‘alert’ to what happens in the outside world.

The leadership needed for implementing and upholding this type of governance does not necessarily start, nor end, at the top of the organization (Ghoshal and Bartlett 1997; Collins 2001). An important trend over time, reflecting Scharmer and Kaufer’s observation, is the shift from leadership at the top of the organization towards more inclusive leadership. This is articulated particularly well in the Robertson’s concept of holacracy or Laloux’ concept of self-management. Both concepts take the ideas of their predecessors to the logical conclusion that responsibility, motivation, meaningful work, business success, and caring for the wider ecosystem are enhanced if the members of the organization are in a position to contribute to and influence organizational decision-making. The concept of self-management is not limited to small and medium-sized enterprises. An interesting example is the Chinese business conglomerate Haier (Frynas et al. 2018) – an assembly of companies with over 80.000 employees. Over its 50-year existence the company has gone through processes of continuous change and growth, led by the idea that teams of employees should be able to direct and control – and are responsible for – the organization of their work processes and their outputs. The latest development is that Haier is creating micro-companies. However, even if organizations are not self-managed the trend towards more inclusive decision-making has become visible.

Another element that strongly characterizes successful companies is that in addition and next to the decentralization of power, they simultaneously organize their processes based on a strong sense of responsibility and discipline. Traditional companies do not differ in this respect from recent currents in management theory (Collins and Porras 1996; Ghoshal and Bartlett 1997; De Geus 1997; Collins 2001). Also, teal, healing, or fair companies (Laloux 2014; Sisodia and Gelb 2019; Bodanis 2020) put emphasis on combining respect and care for their employees, suppliers, business partners, local communities, society at large and the natural environment with order and discipline, be it in different ways then what was common in the past. It also makes sense. If listening is crucial to become a fair organization, it requires structures and an organizational culture to bring it about.

Over time many, if not most, companies have come to understand the relevance of multi-stakeholder management and appreciate the value of it – both for the stakeholders as well as for the financial bottom line of the company itself. As an example of the adoption of stakeholder management, we refer to the nearly 200 CEOs who became signatories to the 2019 Statement on the Purpose of a Corporation of the American Business Roundtable. By signing the statement, the signatories – ranging from Amazon’s Jeff Bezos to Blackrock’s Larry Fink and from Apple’s Tim Cook to Exxon’s Darren Woods – express their commitment to deliver value to all stakeholders for the future success of their companies, their communities, and the country. As the statement concludes: “Each of our stakeholders are essential”.^7^ This means that successful companies are committed to make money, but not as their only or even as their primary objective.

“Yes, they seek profits, but they’re equally guided by a core ideology – core values and sense of purpose beyond just making money”. (Collins and Porras 1996:8)

Unfortunately, however, Wharton professor Tyler Wry^8^ falsified the promise made by many of signatory companies. They laid off 20 percent more employees and returned 20 percent more dividend to shareholders than non-signatories during the Covid-19 pandemic. Apparently, old habits die hard. It demonstrates that a shift towards a more inclusive, just, and sustainable business organization that respects stakeholders and acknowledges their subjectivity, requires more than just an update of the purpose of the corporation or a fancy CSR policy.

#### Collaboration Between Organizational Members

Previously, we observed that business organizations tend to instrumentalize employees, customers, suppliers, the community in which they operate, and nature, based on the logic of having. To paraphrase (but slightly alter) the famous quote by Peter Drucker: leaders eat employees for breakfast. Actually, as representatives of their organizational culture and strategy their breakfast seems to consist of other stakeholders as well – with the exception of the owner of the business. At the same time, it would be an exaggeration to assume a reductionist view and think of all companies treating their stakeholders as ‘it’, purely to satisfy their self-interest and that of their shareholders.

Successful companies go beyond the ego of their leaders. An important part of the grammar of business is to conceive of a business as ‘E pluribus unum’. Organizing refers to operating as a team and collectively working towards a common goal. This focus on ‘Out of many, One’ addresses the importance of the organization’s culture and the values-driven ethical climate of a business. Great leaders, Collins observes, are humble and intensely professional at the same time. However, he continues, “[i]f you only get the humility side, you miss the whole idea”, since they are “infected with an incurable need to produce results” (Collins 2001:30). Leaders create an environment in which employees enjoy freedom and, as a consequence, act empowered and responsibly (Ghoshal and Bartlett 1997:52/53). Laloux even goes as far as to portray CEOs as mere facilitators of organizational processes and leaders by example. Most management authors describe executives and managers as being focused on establishing strong cultures, respecting employees as bearers of the values of the organization. Strong values, De Geus argues, can result in long-lasting organizations. Mitsui Zaibatsu held strong values and principles that were laid down by Mitsui Takatoshi, who founded Mitsui in 1673. After WW II the Zaibatsu was dissolved into 170 independent companies, but in 1952 several of these companies started to form a new keiretsu. Mitsui Bussan was built on a common identity and was “too infused with Mitsui’s values to leave the name and the identity behind” (De Geus 1997:111).

Having values, principles, a strong culture and “a sense of belonging” (De Geus 1997:6) may result in great, living, and lasting companies, it does not guarantee that a company is focused on the well-being and flourishing of others. However, no company survives if it does not live in harmony with its external environment and ignores to adapt to changes in that environment. So, the core question on *who* the right members of the organization are should be complemented with answers to the question on *how* to work together to sufficiently reflect changes in the societal and ecological systems in ways that overcome the instrumentalization of human and non-human stakeholders. It is precisely this challenge that Paul Starrett and his brothers were able to deal with adequately and convincingly. He not only was able to bring the right people together to do the job, but also found ways to motivate them to do the job in time and with sufficient quality. He stood out of the crowd of constructors by seeing his employees as human beings that were giving their best for a challenge that nobody had taken up previously. The values his organization expressed a real-world perspective that sustained the national community of which the organization was part beyond its instrumental value.

In studying the enlightened capitalists in the twentieth and twenty-first century, O’Toole observes that in the end the collaboration between organizational members can only survive over time if the ownership structure allows the company to be in full financial control. Of all the companies he reviewed, only four were able to sustain what could be called a loving environment. Two of these four companies were controlled by family foundations and employees. Two others by trusts, “the boards of which are legally bound to preserve their founders’ values and ensure that employees now and in the future are prime beneficiaries of all profits earned, after reinvestment” (O’Toole 2019:433). In other words, when shareholders are in control of the company it becomes more difficult to pursue or uphold beneficial love compared to companies that are not publicly owned.

#### Organizational Performance

What contribution do successful companies make and for whom? Rather surprisingly, De Geus argues that investment returns for shareholders have “nothing to do with longevity”. Financial accounts describe the past. He continues:

“The profitability of a company was a symptom of corporate health, but not a predictor or determinant of corporate health. (…) [It does] not indicate the underlying conditions that will lead to deteriorating health in the future.” (De Geus 1997:7)

Alternatively, what matters for the longevity of organizations is – a demonstration of – its value in terms of a sensitivity towards the environment, cohesion and identity, tolerance, and an ability to govern its own growth and development through conservative finance. Unfortunately, many business organizations still live by the rule of shareholder capitalism, even those committed to become stakeholder-focused corporations. In order to stay on top as being great, lasting or living, however, organizational performance in the 2020s and beyond requires a focus beyond bringing the right people on board, a strong culture and a mere focus on financial performance as the ultimate measure of success. It is not that the authors that we refer to in this contribution were wrong, as Sisodia and Gelb (2019:26) suggest. The latter authors suggest that, for instance, Collins defines ‘greatness’ only in terms of an outstanding financial performance in comparison to their peer group. They ignore, however, that in order to become a visionary, great, living, or individualized company, the organization and its leadership constantly has to be alert and adapt to changes in its environment. Being successful over time requires executives, managers and employees strongly emphasizing and enacting the core values and mission of their organization, while acknowledging the changes taking place in both the internal and external context in which they operate. It leads them to continuously be geared towards adaptation and change on an operational level. This constant focus on organizational renewal, opens the opportunities for an agapeic turn expressing performance and accountability regarding ‘non-financial capital’ in general, and ‘human capital’ and ‘natural capital’ in particular (Mayer and Roche 2021). What the metrics for the growth of human and natural capital do not sufficiently reflect is the specific focus on beneficial love. Even the most advanced measurement systems do not start with recognizing employees, customers, suppliers, business partners, communities, and nature, by listening to these stakeholders, by acknowledging their subjectivity, and by creating an environment in which they can flourish as ends in themselves and not only as instruments in the hands of management. This even counts for financial stakeholders – who are mostly portrayed one-dimensionally as being only interested in maximizing financial returns, and therefore in businesses maximizing profits.

## Beneficial Love and the Construction of the Empire State Building

Paul Starrett understood that his contracted employees were more than just a pair of hands that could assist in doing the job of constructing the Empire State Building. They were human beings who deserve respect, protection, and being heard, while employing them as craftsman for their instrumental value in the process of construction. He brought the right people together, created a community of dedicated craftsmen based on a set of strong values, listened to their needs and their suggestions, and acted upon their proposals for improvement. Starrett did not produce formal ‘non-financial accounts’, nor did he openly speak too much about his ideas. He also operated within a traditional economic context in which homo economicus was truly influential. Nevertheless, he acknowledged and enacted in his leadership and his organization that self-interest and treating others fairly as subjects were not contradictory, but actually provided an explanation for his success.

The construction of the Empire State Building provides an interesting case of what we have called beneficial love. Not that Paul Starrett was the perfect example of a beneficially loving leader, creating a beneficially loving organization. The perspective of his business activities being part of a wider societal and ecological system (Scharmer and Kaufer 2013) was mainly absent. It was simply not something that was on people’s mind at that time in America just before, during and after the Great Crash. The way he approached his employees, however, does reflect beholding “the other as a Thou rather than an It in order to know what might promote their complete well-being”, as we described as the first element of beneficial love.

How the constituents of business are arranged is shaped by the second element: a living grammar that organizes words so that we can construct proper sentences, communicate effectively, and create a practice that expresses beneficial love in a healthy and productive manner contributing to the good of the other and of the self and unites them. Being an experience constructor, Starrett was far from naïve in treating his employees as subjects that deserve respect for who they are and not just for what they do. As Bodanis (2020:66) observes:

“Starrett has survived years in New York construction, a life-experience which disabuses anyone of belief in the inherent benevolence of mankind.”

It is for this reason, Bodanis continues, that Starrett Brothers Inc. ‘gave, but audited’. The company kept its promises and treated its employees fairly and respectfully for who they were, which was reciprocated by the workers. The brothers “were getting back a lot more than they gave out, but that’s the magic of gratitude” (Bodanis 2020:69).

The third element frames beneficial love as a *latent construct* that emerges when the both grammars combine constituents in a life-affirming, relational way unfolding over time. The story of love is most life-affirming when it promotes the deepest flourishing of all people, living things, and life-sustaining systems. By engaging others with justice, fundamental dignity, and the (re)generation of the intrinsic worth of the other as a persona, Starrett Brothers Inc. created a context for employees, business partners and other stakeholders to flourish and to treat each other as ends in themselves. The construction company created a business environment in which trust prevailed:

“Since the project’s multitude of subcontractors found that they too could depend on what the other said, a powerful form of fast-tracking started up: one of the first for a construction project of this size. Foundries knew they’d been given an honest date by which they’d need to have the first steel beams ready. Likewise, suppliers of elevator cables, and producers of the cement needed for floor pouring, and structural engineers and mechanical engineers and hundreds of other participants could trust what they were being asked to do.” (Bodanis 2020:69)

It leads Bodanis to the conclusion that it was “the irritable, grouchy Starrett who was loved”. It resulted in a gradually evolving chain of mutually respectful and beneficial interactions, leading to a timely opening of the building in 1931.

## An Agenda for the Future of Beneficial Love in Business

The case of Starrett Brothers Inc. is just one of many examples that one can find in the business community. Herb Kelleher, co-founder and former CEO of Southwest Airlines, is a notable contemporary example of leading with love in ways that enhanced, rather than impeded, profitability and organizational effectiveness (Bodanis 2020). In one famous example, when it appeared that the company would have its first unprofitable year in its then 30-year history, Kelleher wrote a letter to employees requesting that they each try to save five dollars per day and he signed the letter, “Love, Herb.” According to one account, his use of the word “love” was “notable because he meant it – and everyone knew it. They remembered all the ways he demonstrated his love over the years… and Southwest had yet another profitable year.” ((Tenney 2014:26) This love is the primary reason why 5,000 Southwest employees attended Kelleher’s memorial service in 2019. (Hall 2019).

A few lessons can be drawn from this contribution. First, we show that beneficial love is not a new kid on the block – not in the management and organizational literature nor in the practice of business organizations. In fact, there is a long history of love and related concepts playing an integral role in leadership in a variety of contexts, including the military. For example, a U.S. Air Force leadership manual written in 1948 repeatedly used the word “love,” as well as “kindness” and “compassion,” in conveying the importance of demonstrating to troops that a leader “is vitally concerned with their welfare.^9^ In other words, that an I-Thou relation is required, and that flourishing of both the individual and the unit is the goal. At that time, the grammar of love was helpful in conveying this message. The contemporary version of the Air Force leadership manual has excised words such as love, opting instead for the language of “strategic” or “tactical” leadership, and thereby reducing the possibility that leaders will create a “container” that is capable of nurturing the kinds of outcomes that are necessary for full flourishing. (Brown 2018).

Returning to the business context, our approach builds on previous research that already highlighted characteristics of successful companies that implemented and displayed aspects of a context in which humans and non-human nature is respected in its own right. These companies started by getting the ‘right’ people on the bus. Once they are on the bus, leaders seriously listen to employees, clients, suppliers, financiers, the local communities, and other relevant stakeholders. It was together that they determined where to drive the bus to – based on values and strong culture.

What agapeic, beneficial love adds to what we know through previous authors is that it creates an environment in which people are to a large extent in control of their work *as part of a dignified life*. They are more than mere resources for the pursuit of corporate success. Viisi provides an excellent example in this respect. The company provides a holacratic decision-making environment in which employees can fully control their work – in collaboration and alignment with the nearest colleagues – and their work-life balance. For example, if they want to take a sabbatical to go skydiving, kitesurfing, hiking in the mountains, take a yoga retreat, or do something else that they consider meaningful, that is all possible. They are seen as grown-up responsible persons able to determine what is good for themselves, their team, and the organization at large. In return for this gift of trust by Viisi and their colleagues, they reciprocate through motivation, helpfulness, and a desire to produce excellent results for the company. The score for employee satisfaction on a scale from 1–10 exceeds 9.5 just like the score for client satisfaction, which also transcends 9.5. The company had no problem whatsoever to finance the growth of the company. Within a limited timeframe it obtained the loan capital through an online crowdfunding campaign at an interest rate below bank financing.

Second, more than simply transcending the false choice between beneficial love and profitability, bringing the grammars of love and business into greater dialogue holds great promise for reimagining business as a platform for solving social problems, contributing to (re)generative environments, and promoting the good society. Research has found that expression of love in a long-term care facility made a positive difference in the lives of workers, clients, and families of clients (Barsade and O’Neill 2014). Addressing a broader range of issues, Laloux’s *Reinventing Organizations*, Sisodia & Gelb’s *The Healing Organization*, Chapman & Sisodia’s *Everybody Matters*, Mackey & Sisodia’s *Conscious Capitalism*, Scharmer and Kaufer’s *Leading from the Emerging Future*, and many other management books provide dozens of examples of companies addressing the need for more sustainable, just, inclusive and loving business. For example, Chapman’s leadership of Barry-Wehmiller, a collection of companies in the global manufacturing technology industry, prioritizes nurturing the growth of people rather than building things. As Chapman puts it, “We’re in business so that all our team members can have meaningful and fulfilling lives…. In other words, Barry-Wehmiller is in business to improve lives.” (Chapman and Sisodia 2015:69) As Barry-Wehmiller’s track record demonstrates, prioritizing the care of people is consistent with financial success. But despite such case studies, and indeed the good quality of much of the current business literature, additional systematic research is needed into the practice of love in business and how to best promote and support such practice in financially and operationally sustainable ways. More best practices are required that show that the grammar of love is not contradictory to the grammar of business.

Much of this work will involve engagement with the full range of human motivations in business setting, which is quite familiar territory in the management literature (McGregor 1960; Ouchi and Price 1978; Guillen 2021) But there is a need for fresh perspectives as well. One promising development is the emergent awareness of the importance of the capabilities of individuals and groups to express beneficial love in action, based on everyday practices like help offering, collective decision making, and solving problems directly (Lilius et al. 2011). Beyond the individual willingness to contribute to the flourishing and well-being of others, teams, work groups, and entire organizations can build the reliable capacity needed to ensure that the needs of others (Ignatieff 1984) will be both noticed and addressed effectively, and also that this labor will be distributed equitably throughout the organization. Preliminary research suggests that such a climate also promotes complete well-being, or full flourishing, which is an important outcome of both love and humanistic management.

Third, more organizations better express the commitment to the well-being of all stakeholders as subjects – as sacred Thou’s – in their search for profit. We fail in striking this balance to the extent that we start with the premise that self-interest, care for others, and care for the planet are mutually exclusive pursuits. (Sisodia and Gelb 2019:19) In fact, high-performing individuals and organizations assume abundance rather than scarcity and they manifest this in the vibrant relationships that they co-create with others and with the natural world. (Ritchie-Dunham 2014) Research in the business setting on striking the balance between profit and what we have labeled AGAPE – the Agenda for Growth and Affirmation of People and the Environment – is in its infancy as an integrated field of study, but some holistic frameworks are catching on (Scharmer and Kaufer 2013; Raworth 2017). One helpful path forward is to compile and synthesize the lived experiences of leaders and organizations who have integrated the grammars of love and business in order to inductively develop a theory of change that might help other early adopters and followers make the leap. The potential for learning is enhanced by creating a space for executives, managers, and employees to share with each other – both inside and outside their own organizations – the challenges they have faced, the lessons they have learned, and their successes with scaling beneficial love in their teams, units and organizations and beyond. As we have stated, the dialectic of love and its opposites are ever-present in businesses. Few leaders seek the mantle of an unloving and uncaring culture and behavior and virtually all would like to be thought of as promoting flourishing. The grammar of love can help bring into focus the comparative health and well-being of a business culture and its extractive or (re)generational relationship to the broader world.

Fourth, and somewhat contrary to one of the latest currents in the management literature, including Simon Sinek’s ‘Start with Why’ (2010) or Aaron Hurst’s ‘The Purpose Economy’ (2019), most scholars that previously focused on the right balance between a humane and respectful organization on the one hand and a successful one on the other, are agnostic about the ‘what’ and the ‘why’ of the business. It is the ‘who’ and the ‘how’ that determine and preserve business success over the long run. The quality of any system, Scharmer and Kaufer argue, “depends on the quality of awareness from which people in the system operate” (Scharmer and Kaufer 2013:18). This awareness progressively comprises the needs and interests of a wider community of stakeholders – including our planet and future generations (Krznaric 2020).

We conclude with the recognition that the status quo of businesses exploiting humans and non-humans – including the natural system in which businesses operate – is both unsustainable and undesirable. Research suggests that many people are languishing – only 17% are “flourishing”. The vast majority of employees are not engaged at work, and that many of the planet’s life-support systems are in serious decline (Keyes 2002; Wigert et al. 2021). Whatever word we might use to describe this state of affairs, it is clear that “love” is not a contender. Further integration of the grammars of love and business is necessary for progress. We have suggested that this involves the alignment of business goals, culture, decision-making, outputs, and outcomes with the promotion of flourishing of all people, living things, and life-sustaining systems. Beneficial love, leading to human and ecological flourishing, is not the only ingredient, but it is an important and often overlooked one. No organization can survive in the long-term without a harmonious relationship with the broader social and natural environment. In too many instances, the love/hate dialectic has been tipped in the wrong direction – or resulted in indifference at best. Extractive relationships with the social and natural environment have characterized many business operations, but as we have shown, there are many encouraging examples to the contrary. We invite business scholars, executives, managers, and employers, as well as others dealing with or experiences the consequences of businesses behavior, to advance the dialogue about how to conduct business in the spirit of beneficial love.

## Footnotes

1 In contrast: the construction works for the World Cup Soccer in Qatar took more than 6,500 (most migrant) workers’ lives (https://www.theguardian.com/global-development/2021/feb/23/revealed-migrant-worker-deaths-qatar-fifa-world-cup-2022).

2 Often, when speaking of love, distinctive Greek words are used such as the four loves according to C.S. Lewis: storge, philia, eros and agape. When we use the word love in a business context, we primarily refer to the concept of agape.

3 By ‘totalization’ Levinas means that we cannot know the world outside ourselves independent of our own senses and thoughts. Humans are always at the center of their own totalizing: they make the world to their own personal world through observing, acting and giving meaning. Totalization is not an act that is subject to our will. We cannot simply choose to do it or leave it behind. My totalizing is inevitable, and nothing falls outside of this totalizing grasp.

4 Starrett started his professional career in 1988 working for Chicago-based architects Daniel Burnham and John Wellborn Root – at first as a stenographer. In 1897 he began to work for George A. Fuller Company, a construction company in Chicago that opened offices in New York in 1900 and made Paul responsible for all projects in Manhatten north of Fourteenth Street.

5 The seventy-story Bank of Manhattan Building (1929–1930) at 40 Wall Street had been a key to his obtaining the Empire State job because construction speed was so important: the bank building went up in eleven months by starting the new foundations while the old buildings were still being demolished above (Tauranac 1995).

6 Deliberately, we do not speak of ‘the loving company’ as it would objectify and reify a company as being made up of love (whatever that is), instead of a company in which people are respecting and listening to each other and which continuously seeks to establish an environment and a culture that allows individuals and collectives to grow, develop and flourish.

7 https://opportunity.businessroundtable.org/ourcommitment/

8 https://www.theatlantic.com/ideas/archive/2020/08/companies-stand-solidarity-are-licensing-themselves-discriminate/614947/

9 Department of the Air Force. (1948). Air Force Leadership (AFM 35–15). Department of the Air Force.
